# Obesity and pregnancy: a transversal study from a low-risk maternity

**DOI:** 10.1186/1471-2393-14-249

**Published:** 2014-07-28

**Authors:** Ana Carolina S Calderon, Silvana M Quintana, Alessandra C Marcolin, Aderson T Berezowski, Luiz Gustavo O Brito, Geraldo Duarte, Ricardo C Cavalli

**Affiliations:** Department of Gynecology and Obstetrics, Ribeirão Preto School of Medicine, University of São Paulo (FMRP-USP), Avenida Bandeirantes, 3900 8th floor, Ribeirão Preto, 14049-900 SP Monte Alegre Brazil

**Keywords:** Obesity, Pregnancy, Perinatal care, Induction of labour

## Abstract

**Background:**

Obesity is a public health problem and is increasing in all populations, including pregnant women. It influences maternal and neonatal outcomes; however, data are scarce in developing countries. We aimed to compare perinatal results between obese and non-obese pregnant women in a low-risk maternity.

**Methods:**

Transversal study of 1,779 40-week-pregnancies from 2005 to 2009 that completed a standard questionnaire with sociodemographic, obstetrical and neonatal variables and performed an ultrasound with amniotic fluid index (AFI) measurement and foetal vitality (FBP, non-stress test). They were analysed about their association with obesity on pregnancy.

**Results:**

When compared with non-obese women, the group of obese patients had higher systolic (118.1 vs 109.2 mmHg; p < 0.01) and diastolic (76.6 vs 70.4 mmHg; p < 0.01) pressure levels, AFI (12.52 vs. 9.61 cm; p = 0.02), presence of meconium on labour (20.52 vs. 14.67%; p = 0.02), birthweight (3602 vs. 3437 g; p < 0.01) and caesarean section (39.74 vs. 29.98%, p < 0.01).

**Conclusions:**

Labour induction before 40 weeks in the antenatal period associated with foetal weight estimation should be considered as a recommendation for decreasing high percentages of caesarean delivery found in obese women.

**Electronic supplementary material:**

The online version of this article (doi:10.1186/1471-2393-14-249) contains supplementary material, which is available to authorized users.

## Background

Obesity constitutes a major public health issue, and can be characterized as an epidemic, that does not discriminate gender, age or socioeconomic level [1]. Diagnosis is commonly performed by calculation of Body Mass Index (BMI), whose values above 30 kg/m^2^ are present between 15 and 20% of global population [1]. However, one group catches our attention among the obese population: women of reproductive age, because it is increasingly more frequent for this population to become pregnant above the recommended weight. When we compare this group to men with the same age or women at older ages, the former has a large prevalence of obesity [1].

In the United States, the last NHANES (National Health and Nutrition Examination Survey) found that 26% of non-pregnant women between 20 and 39 years were overweight and 29% were obese from 1999 to 2002 [2]. In Australia, approximately 15% of women between 25 and 34 years were obese between 2004 and 2005 [3], a lower percentage when compared to the United Kingdom, where reports of 32% of overweight and 20% of obesity were found among women from 16 to 64 years [4]. Almost 40% of married women were obese in the United Arab Emirates [5]; in Denmark, the percentage of obese women grew from 3.1 to 7.8% in a ten-year analysis [6].

Nevertheless, not only women become pregnant overweight but also gain additional weight during gestation. A study found that obesity increased from 9.9% in 1990 to 16% in 2004 among pregnant women in the North East of England [7]. An assessment performed among developing countries has ascertained that, in thirty-six of them, the proportion of overweight and obese women was higher than that of recommended weight among women, especially on urban population [8].

According to the Institute of Medicine (IOM) and the National Heart, Lung, and Blood Institute of the National Institutes of Health, the recommended weight gain for obese women during pregnancy is up to 6.8 kilograms; for overweight women, gain would be from 6.8 to 11.2 kilos and for non-obese women, between 11.2 to 15.9 kilos [9]. In the United States, it was shown that one in three pregnant women will fit in these prerequisites for weight gain during pregnancy, according to PRAMS (Pregnancy Risk Assessment Monitoring System) data bank [9]. In Switzerland, it was observed that 14.2% of pregnant women gained more than 20 kilos during gestation in 2004 against a percentage of 2.6% in 1986 [10].

The excessive weight gain during pregnancy and obesity before gestation is related to higher morbidity and mortality. Among possible complications, it can be cited: abortion, congenital anomalies, thromboembolism, hypertensive disorders (gestational hypertension, preeclampsia, eclampsia and HELLP syndrome), gestational diabetes, prematurity, macrosomia, foetal death, higher percentages of anaesthetic risk, failure of labour induction and as consequence, higher indexes of caesarean section, dystocic labour, postpartum infection and haemorrhage [1, 6, 11–14].

Prolonged pregnancy is another theme that deserves attention, since perinatal morbidity and mortality are slightly increased in gestations over 40 weeks if foetal assessment is not correctly performed [15]. Main risk factors that can be associated with prolonged pregnancy are related to low socioeconomic level of women, excessive weight gain during pregnancy and previous prolonged pregnancy [16]. Perinatal complications associated with prolonged gestations are foetal macrosomia, placental dysfunction, acute foetal distress, oligohydramnios, meconium emission and aspiration and perinatal death [15].

The present study aims to assess the perinatal results from a cohort of pregnant obese women with gestational age over 40 weeks compared to non-obese patients from a low-risk maternity in a municipality from Southeastern Brazil.

## Methods

A transversal study was undertaken at Centro de Referência à Saúde da Mulher – CRSM-MATER, between 2005 and 2009, with consecutive sampling of 1,780 patients that were receiving prenatal assistance and would be forwarded to this hospital when gestation resolution arise. This research was approved by the Institutional Reviews Board from Hospital das Clínicas da FMRP-USP and CRSM-MATER and followed the STROBE protocol for observational studies (Additional file 1). Informed consent was obtained from all participants from the study. We did not have any patients that declined to participate.

The inclusion criteria were: women with 40 weeks’ gestation calculated from amenorrhoea period and/or first-trimester ultrasound; singleton foetus with cephalic presentation; women with no comorbidities or gestational intercurrences. Exclusion criteria were: gestational diabetes, maternal heart diseases, changes in foetal morphology found by ultrasound, other factors that could attribute risk to current gestation, hypertensive disorders.

After 40 weeks or more, patients were clinically evaluated by non-stress test and ultrasound according to the institution protocol: prenatal consultation at 40 and 41 weeks’ gestation, with further reassessments every three days (41 weeks + 3 days, 41 weeks + 6 days, 42 weeks) for reassessment; when gestational age (GA) was 42 weeks, pregnancy resolution was scheduled by labour induction. To elaborate this research, only data from the evaluation performed at 40 weeks gestation was used.

Antenatal cardiotocography (Toitu MT-325, Tokyo, Japan) was undertaken after the patient had an adequate diet and was positioned at horizontal decubitus with a 45° angle on the headboard in a separate room. Foetal heart rate (FHR) tracings were obtained for 20 minutes with no uterine contractions and if the foetus was hypoactive or needed stimulus, a vibratory or sound stimulus was performed and FHR was recorded for more three minutes.

Foetal biophysical profile (FBP) was performed using a 5-2 MHz convex probe from a Logiq 100 Pro ultrasound (General Electric, United Kingdom) with assessment after an adequate diet with the patient positioned at horizontal decubitus. Foetal observation was done in thirty minutes to register foetal breathing movements (at least one episode of ≥ 30 seconds), gross body movements (at least three episodes body/limb movements), foetal tone (at least one episode of active extension with return to flexion), reactive FHR (at least two episodes of acceleration with foetal movements) and amniotic fluid index (AFI) (at least one pocket of fluid measuring ≥2 cm in vertical axis), as described by Magann et al. [17]. Amniotic fluid index (AFI) was assessed using the classification proposed by Phelan, where the abdomen is divided into four quadrants, with the umbilicus delineating the upper and lower halves. The deepest, unobstructed, vertical pocket of fluid is measured in each quadrant in centimeters. The four pocket measurements are then added to calculate the AFI [17].

Dependent variables were: sociodemographic (age, race, marital status, professional activity, educational level), obstetrical (number of gestations, births and abortions, tobacco and/or alcohol use, drug addiction, systolic and diastolic blood pressure, number of prenatal consultations, cardiotocography report with 40 weeks, type of delivery (vaginal or caesarean), Bishop index, placental maturation grade, FBP, presence of ruptured membranes) and neonatal (new born weight and gender, presence of foetal meconium). The independent variable was the presence or absence of obesity, defined as a BMI equal to or over 35 kg/m^2^.

For statistical analysis, continuous variables were expressed by means and analysed by t-Student test. Binomial variables were analysed by chi-square and Fisher tests. A significance level of 5% was stipulated. Missing data was treated as completely at random and used treatment was listwise deletion. Data were stored into a Microsoft Excel spreadsheet and statistics were calculated in the SPSS software (SPSS Inc., Chicago, IL, USA).

## Results

Mean BMI from obese and non-obese were, respectively, 38.76 kg/m^2^ and 25.98 kg/m^2^. Tables 1 and 2 showed the results obtained after the comparison between obese and non-obese population. We can notice that obese pregnant women presented higher systolic and diastolic blood pressure levels than non-obese patients; however, basal blood pressure levels of obese women were below the minimum required for diagnosis of arterial hypertension.

**Table 1:** **Obstetric and perinatal variables and their relation with obesity during pregnancy**

Variables	Obese (BMI > 30 kg/m ^2^)			Control (BMI < 30 kg/m ^2^)				
	n	Mean	SD	n	Mean	SD	p*	Total (n)
Age (years)	222	25.47	5.29	1,544	24.11	5.41	<0.01	1,766
Age at first pregnancy (years)	221	20.76	4.82	1,543	20.36	4.42	0.21	1,764
Previous pregnancies	228	2.29	1.51	1,550	2.03	1.39	0.01	1,778
Previous abortions	228	0.52	0.98	1,551	0.21	0.52	<0.01	1,779
Previous vaginal deliveries	228	0.64	1.20	1,551	0.68	1.15	0.71	1,779
Previous caesarean deliveries	228	0.23	0.42	1,551	0.16	0.37	0.01	1,779
Gestational age at 1^st^ US (weeks)	127	15.11	4.74	1,273	15.09	4.71	0.96	1,400
Pre-natal attendance**	91	8.90	1.55	761	8.83	1.73	0.70	852
Systolic blood pressure (40^th^ week)**	93	118.1	12.6	773	109.2	11.5	<0.01	866
Diastolic blood pressure (40^th^ week)**	93	76.6	9.9	773	70.4	8.6	<0.01	866
Bishop index (40^th^ week)**	93	1.88	1.47	773	2.21	1.80	0.09	866
FBP (40^th^ week)	228	7.89	0.49	1,538	7.92	0.38	0.31	1,766
AFI (40^th^ week)	228	12.52	4.36	1,539	9.61	3.57	0.02	1,767
Placenta (40^th^ week)**	93	2.13	0.54	777	2.19	0.55	0.32	870
Gestational age during birth (weeks)	229	40.84	0.71	1,551	40.75	0.66	0.06	1,780
Birthweight (g)	229	3,602	470	1,551	3,437	414	<0,01	1,780
Apgar 1^st^ minute	229	7,76	2,00	1,551	7,90	2,06	0,33	1,780
Apgar 5^th^ minute	229	9,53	0,65	1,551	9,59	0,78	0,29	1,780

*FBP* – Foetal biophysical profile.

*AFI* – amniotic fluid index.

*Statistical significance when p < 0.05 – T Student test.

**Sampling less than 1,000 patients.

**Table 2:** **Maternal and neonatal characteristics and their relation with obesity during pregnancy**

Variable	Obese (n)	%	Non-obese (n)	%	p
**Color**					0.69
White	66	70.97	533	68.95	
Mulatto	17	18.28	132	17.08	
Black	10	10.75	108	13.97	
**Marital status**					0.42
Without partner	18	19.35	186	24.35	
With partner	75	80.65	578	75.65	
**Professional activity**					0.15
Without	42	45.16	290	37.52	
With	51	54.84	483	62.48	
**Scholarity (years)**					0.98
0-4	26	27.96	199	25.75	
5-8	33	35.48	353	36.48	
>8	34	36.56	292	37.78	
**Tobacco use**					0.66
No	203	88.65	1,358	87.61	
Yes	26	11.35	192	12.39	
**Alcohol use**					0.78
No	223	97.38	1,515	97.68	
Yes	6	2.62	36	2.32	
**Ilicit drug use**					0.52
No	228	99.56	1,358	99.16	
Yes	1	0.44	13	0.84	
**Cardiotocography**					0.35
Active	211	92.54	1,374	89.45	
Hypoactive and reactive	15	6.58	146	9.51	
Hypoactive and hyporreactive	2	0.88	16	1.04	
**Type of delivery**					<0.05*
Vaginal	138	60.26	1,086	70.02	
Caesarean	91	39.74	465	29.98	
**Ruptured membranes**					0.32
No	193	84.28	1,265	81.56	
Yes	36	15.72	286	18.44	
**Sex of newborn**					0.78
Female	116	50.66	770	49.68	
Male	113	49.34	780	50.32	
**Presence of meconium**					0.02*
No	182	79.48	1,320	85.33	
Yes	47	20.52	227	14.67	

*p < 0.05 – Chi-square test.

Ultrasonographic measurement of AFI reported higher means for obese women than controls (12.52 vs. 9.61; p = 0.02). Another variable that showed a significant difference between these two groups was birth weight, which was higher in obese women (3602+/-470 g vs. 3437+/-414 g; p < 0.01).

About types of birth, caesarean percentages were significantly higher in obese patients than controls (39.74% vs. 29.98%) and it was found a statistical association with macrossomic foetuses (p < 0.05). Obese women were also more prone to induce labour than non-obese patients, as shown in Table 3 (p < 0.01).

**Table 3:** **Labour induction with obese and non-obese women**

	Induction	Proportion	CI 95%
**Non obese**	531/1427	37.21%	34.74 - 39.75
**Obese**	100/211	47.39%	40.76 – 54.12

(p < 0.01; Fisher test).

Non-obese women presented higher incidence of ruptured membranes previous to admission. However, obese patients reported with more frequency in the presence of meconium during labour.

## Discussion

Our study found that obese women have a higher risk of having their birth induced, heavier babies and more susceptible to caesarean section, in agreement with the available literature. About labour induction, our data are important to discuss the influence of obesity on birthweight during prenatal counselling and, as a consequence, in obstetrical outcomes, such as the type of birth. One study found a success rate of 60% in vaginal delivery for primiparous women and 90% for multiparous women [3].

A retrospective study including 287,213 pregnancies in London, with 176,923 non-obese women and 31,273 obese patients has demonstrated a rate of labor induction of 15.26% in the former group, and of 24.65% in the latter group (OR 1.70 [1.64-1.76] vs. 2.14 [1.85-2.47], CI 99%). Moreover, the study reported higher birthweight indexes and elective/emergency caesarean section rates for obese women [18]. Another cohort by Arrowsmith et al. with 29,224 pregnancies showed an increase in labour induction rates proportionally related to BMI; obese women had a higher rate of labour induction (34.4%) than non-obese patients (26.2%). Elective and emergency caesarean rates were also higher in obese women, as well as macrosomic foetuses [19].

One hypothesis for the increase in birthweight among obese women is the altered placental secretion of adipocins such as leptin and insulin, as well as an increase on concentrations of free IGF-1. All of them are important mediators of foetal growth, causing the increase at the offer of nutrients to the foetus by: direct increase on expression and activity of transporter proteins at the placental barrier; increased offer of nutrients on maternal circulation [20]. The exacerbated fetal growth observed among obese mothers may be an explanation for the increase in caesarean and induction rates in this group.

Blood pressure was a variable already investigated in the literature. Athukorala et al. studied 1,661 pregnancies and from this sampling, 272 were obese women, showed a statistically significant increase on mean of diastolic (DBP) and systolic blood pressure (SBP) (mean difference of 7.8 mmHg at SBP and 6.1 mmHg at DBP), on induction rates (relative risk of 1.78; 95% CI 1.51-2.09) and caesarean rates (RR = 1.63; 95% CI 1.34-1.99) at obese pregnants, and that the mean birthweight among obese mothers was higher than that observed among patients with a normal BMI (mean difference of 99.7 g with obese patients; 95%CI 21.3-178.2, p = 0.01) [3]. Although an association between obesity during pregnancy and hypertension was verified, we do not believe that basal increase of pressoric levels can predispose the development of hypertensive syndromes in pregnancy.

The presence of meconium before labour and preterm rupture of membranes was associated with obesity. However, obese women did not present lower indices of 1^st^ minute Apgar when compared to non-obese women and this indicates that the presence of meconium would not be associated with worse foetal well being in this group. The association between these two variables (meconium elimination and obesity) has not been adequately studied in the literature, and their stratification with multivariate analysis to eliminate confounding bias reinforces further investigations.

Limitations of the study were found: a multivariate analysis was not performed to decrease confounding bias among variables to determine which would be associated with obesity. However, bivariate analysis could identify major potential factors related to the main outcome. Another limitation is related to missing data. Maybe some variables would have different results using other type of treatments such as data imputation; however, we believe that these results would not be so different considering the similarity of our results with other studies.

## Conclusion

We believe that prenatal counselling before completing 40 weeks of pregnancy associated with an estimate of birthweight through ultrasound fetal biometry may help in the decision of inducing labour in obese women. It may influence on obstetrical outcomes, reducing caesarean rates and minimizing post procedure complications.

## Electronic supplementary material

Additional file 1: **STROBE Statement: STrengthening the Reporting of OBservational studies in Epidemiology.** (DOC 84 KB)

## Abbreviations

AFI: Amniotic fluid index
BMI: Body mass index
DBP: Diastolic blood pressure
FBP: Foetal biophysical profile
FHR: Foetal heart rate
IOM: Institute of Medicine
NHANES: National Health and Nutrition Examination Survey
NST: Non-stress test
PRAMS: Pregnancy Risk Assessment Monitoring System
STROBE: STrengthening the Reporting of OBservational studies in Epidemiology.
