# Supplementary material for: Obesity and pregnancy: a transversal study from a low-risk maternity
Source: BMC Pregnancy Childbirth. 2014 Jul 28;14:249. doi: 10.1186/1471-2393-14-249 (PMC4124168; doi:10.1186/1471-2393-14-249)
Supplement: Supplementary file 1 — Additional file 1: STROBE Statement: STrengthening the Reporting of OBservational studies in Epidemiology. (DOC 84 KB) [file 12884_2014_1120_MOESM1_ESM.doc]

STROBE Statement—checklist of items that should be included in reports of observational studies

|  | Item No | Recommendation |
| --- | --- | --- |
| **Title and abstract** | 1 | (*a*) Indicate the study’s design with a commonly used term in the title or the abstract OK |
|  |  | (*b*) Provide in the abstract an informative and balanced summary of what was done and what was found OK |
| Introduction | | |
| Background/rationale | 2 | Explain the scientific background and rationale for the investigation being reportedOK |
| Objectives | 3 | State specific objectives, including any prespecified hypothesesOK |
| Methods | | |
| Study design | 4 | Present key elements of study design early in the paper OK |
| Setting | 5 | Describe the setting, locations, and relevant dates, including periods of recruitment, exposure, follow-up, and data collection OK |
| Participants | 6 | (*a*) *Cohort study*—Give the eligibility criteria, and the sources and methods of selection of participants. Describe methods of follow-up  *Case-control study*—Give the eligibility criteria, and the sources and methods of case ascertainment and control selection. Give the rationale for the choice of cases and controls  *Cross-sectional study*—Give the eligibility criteria, and the sources and methods of selection of participants OK |
|  |  | (*b*) *Cohort study*—For matched studies, give matching criteria and number of exposed and unexposed  *Case-control study*—For matched studies, give matching criteria and the number of controls per case |
| Variables | 7 | Clearly define all outcomes, exposures, predictors, potential confounders, and effect modifiers. Give diagnostic criteria, if applicable OK |
| Data sources/ measurement | 8* | For each variable of interest, give sources of data and details of methods of assessment (measurement). Describe comparability of assessment methods if there is more than one group OK |
| Bias | 9 | Describe any efforts to address potential sources of bias OK – included explanation about missing data |
| Study size | 10 | Explain how the study size was arrived at OK – we decided not to make a sampling calculation, because our sampling was big enough to take our conclusions. |
| Quantitative variables | 11 | Explain how quantitative variables were handled in the analyses. If applicable, describe which groupings were chosen and why OK |
| Statistical methods | 12 | (*a*) Describe all statistical methods, including those used to control for confounding OK |
|  |  | (*b*) Describe any methods used to examine subgroups and interactionsOK |
|  |  | (*c*) Explain how missing data were addressed OK |
|  |  | (*d*) *Cohort study*—If applicable, explain how loss to follow-up was addressed  *Case-control study*—If applicable, explain how matching of cases and controls was addressed  *Cross-sectional study*—If applicable, describe analytical methods taking account of sampling strategy – not applicable |
|  |  | (*e*) Describe any sensitivity analysesOK |

| Results | | |
| --- | --- | --- |
| Participants | 13* | (a) Report numbers of individuals at each stage of study—eg numbers potentially eligible, examined for eligibility, confirmed eligible, included in the study, completing follow-up, and analysed OK |
|  |  | (b) Give reasons for non-participation at each stage – no cases OK |
|  |  | (c) Consider use of a flow diagram – not needed, because no decline cases were presented |
| Descriptive data | 14* | (a) Give characteristics of study participants (eg demographic, clinical, social) and information on exposures and potential confounders OK |
|  |  | (b) Indicate number of participants with missing data for each variable of interest – pointed out in the table OK |
|  |  | (c) *Cohort study*—Summarise follow-up time (eg, average and total amount) |
| Outcome data | 15* | *Cohort study*—Report numbers of outcome events or summary measures over time |
|  |  | *Case-control study—*Report numbers in each exposure category, or summary measures of exposure |
|  |  | *Cross-sectional study—*Report numbers of outcome events or summary measures OK – at the table |
| Main results | 16 | (*a*) Give unadjusted estimates and, if applicable, confounder-adjusted estimates and their precision (eg, 95% confidence interval). Make clear which confounders were adjusted for and why they were included OK – at the table |
|  |  | (*b*) Report category boundaries when continuous variables were categorized OK |
|  |  | (*c*) If relevant, consider translating estimates of relative risk into absolute risk for a meaningful time period OK |
| Other analyses | 17 | Report other analyses done—eg analyses of subgroups and interactions, and sensitivity analyses – not applicable to this study |
| Discussion | | |
| Key results | 18 | Summarise key results with reference to study objectivesOK |
| Limitations | 19 | Discuss limitations of the study, taking into account sources of potential bias or imprecision. Discuss both direction and magnitude of any potential biasOK |
| Interpretation | 20 | Give a cautious overall interpretation of results considering objectives, limitations, multiplicity of analyses, results from similar studies, and other relevant evidence OK |
| Generalisability | 21 | Discuss the generalisability (external validity) of the study results OK – at the conclusions |
| Other information | | |
| Funding | 22 | Give the source of funding and the role of the funders for the present study and, if applicable, for the original study on which the present article is based OK |

*Give information separately for cases and controls in case-control studies and, if applicable, for exposed and unexposed groups in cohort and cross-sectional studies.

**Note:** An Explanation and Elaboration article discusses each checklist item and gives methodological background and published examples of transparent reporting. The STROBE checklist is best used in conjunction with this article (freely available on the Web sites of PLoS Medicine at http://www.plosmedicine.org/, Annals of Internal Medicine at http://www.annals.org/, and Epidemiology at http://www.epidem.com/). Information on the STROBE Initiative is available at www.strobe-statement.org.
